# Preoperative sedentary behavior is neither a risk factor for perioperative neurocognitive disorders nor associated with an increase in peripheral inflammation, a prospective observational cohort study

**DOI:** 10.1186/s12871-020-01200-w

**Published:** 2020-11-14

**Authors:** Sarah Saxena, Christopher Rodts, Vincent Nuyens, Juliette Lazaron, Victoria Sosnowski, Franck Verdonk, Laurence Seidel, Adelin Albert, Jean Boogaerts, Veronique Kruys, Mervyn Maze, Joseph Vamecq

**Affiliations:** 1 Department of Anesthesia, University Hospital Center (CHU de Charleroi), Charleroi, Belgium; 2 Department of Anesthesia and Perioperative Care, Center for Cerebrovascular Research, UCSF, San Francisco, CA USA; 3 Laboratory of Experimental Medicine (ULB unit 222), University Hospital Center (CHU de Charleroi), Charleroi, Belgium; 4 Department of Anesthesiology, Perioperative and Pain Medicine, Stanford University School of Medicine, Stanford, CA USA; 5 Department of Biostatistics, University Hospital of Liège, Liège, Belgium; 6 Laboratory of Molecular Biology of the Gene, Department of Molecular Biology, ULB Immunology Research Center (UIRC), Free University of Brussels (ULB), Gosselies, Belgium; 7 Inserm, CHU Lille, Univ Lille, Department of Biochemistry and Molecular Biology, Laboratory of Hormonology, Metabolism-Nutrition & Oncology (HMNO), Center of Biology and Pathology (CBP) Pierre-Marie Degand, CHRU Lille, EA 7364 RADEME, University of North France, Lille, France

**Keywords:** Inflammation, Perioperative neurocognitive disorders, Cognition, Interleukin-6, High molecular group box 1

## Abstract

**Background:**

Surgical interventions result in a postoperative rise in circulating inflammatory cytokines and high molecular group box protein 1 (HMGB1). Herein, the impact of a sedentary lifestyle and other age-related factors on the development of perioperative neurocognitive disorders (PND) following non-cardiac surgical procedures was assessed in an older (55–75 years-old) surgical population.

**Methods:**

Prior to surgery, patients were asked questions regarding their sedentary behavior and daily habits. They also passed the Mini Mental State Examination (MMSE) and their blood circulating interleukin 6 (IL-6) and HMGB1 levels were assayed by ELISA. IL-6 and HMGB1 measurements were repeated respectively 6 and 24 h after surgery. MMSE was re-evaluated 6 weeks and whenever possible 3 months after surgery.

**Results:**

Thirty-eight patients were enrolled in the study from January until July 2019. The study identified self-sufficiency, multilinguism, and overall health score on the geriatric depression scale, as protectors against PND. No other demographic (age, sex), environmental (solitary/non-solitary housing, professional and physical activities, smoking, alcohol drinking), comorbidity (antipsychotic drug uptake, diabetic state) and type of surgery (orthopedic, general, genitourinary) influenced the development of PND. Although some factors (surgery type and age) influenced the surgery-induced rise in the circulating IL-6 levels, they did not impact HMGB1.

**Conclusion:**

Inflammaging, reflected by the greater increment of surgery-induced IL-6 in patients with advanced age, was present. As trauma-induced release of HMGB1 was not similarly affected by age, we surmise that HMGB1, rather than circulating cytokines, is the key driver of the trauma-induced inflammatory cascade leading to PND.

**Trial registration:**

Clinicaltrials.gov identifier: NCT03805685.

## Background

Perioperative neurocognitive dysfunction (PND) was first described in 1887 [1, 2]. PND remains an important often under-diagnosed, surgical complication that is associated with increased mortality, risk of leaving the labor market prematurely, and dependency on unemployment/disability payments [2, 3]. The etiology of PND is not fully clarified although the type of anesthetic [4], intra-operative physiological perturbations (especially, hypotension and hypoxemia) [5] and the depth of sedation/anesthesia [6] have been advocated and rejected as causally-related. Over the last decade, several studies have suggested that the trauma-induced inflammatory cascade is a key pathogenic mechanism for the development of PND [7–9].

During surgery under general anesthesia the high molecular group box protein 1 (HMGB1) is released into the circulation from traumatized tissues [7]. This damage-associated molecular pattern (DAMP) binds to pattern recognition receptors on circulating bone marrow-derived monocytes (BM-DMs), hence triggering the nuclear translocation of the transcription factor NF-κB which activates gene expression and release of pro-inflammatory cytokines including IL-6 and IL-1β [7]. The onset of this inflammatory state disrupts the blood brain barrier [8]. Within the brain parenchyma the chemokine MCP-1 (also referred to as CCL2) is upregulated and, by signaling through its receptor CCR2, attracts the BM-DMs [9]. The influx of BM-DMs activates the resident quiescent microglia. Together, BM-DMs and activated microglia release HMGB1, IL-6 and IL-1β, thereby disrupting long-term potentiation and the synaptic plasticity involved in cognitive functions of learning and memory [10–12]. Inability to successfully resolve the inflammatory cascade promotes the development of PND [13–15].

Several risk factors have been reported for PND including middle and advanced age and metabolic syndrome when inflammation resolution is retarded [16–18]. As sedentary lifestyle has also been associated with poorly-resolved inflammation [19, 20], we aimed to investigate the impact of sedentary behavior of elderly surgical patient on inflammation (evaluated by circulating IL-6 and HMGB1 levels) and PND (evaluated by MMSE 6 weeks postoperatively).

## Methods

### Patient enrollment and ethics

This prospective, non-controlled, observational cohort study adhered to the Declaration of Helsinki and the STROBE checklist and was approved by the internal review board (ethical committee of the “Intercommunale de Santé Publique du Pays de Charleroi-OM008”). Written informed consent was obtained from each patient enrolled in the study. The trial was registered on clinicaltrials.gov (NCT03805685) (https://clinicaltrials.gov/ct2/show/NCT03805685?term=NCT03805685&draw=2&rank=1) and conducted at the University Hospital of Charleroi, Charleroi, Belgium between January and August 2019.

### Inclusion/exclusion criteria

Inclusion criteria were surgical patients, of both sexes aged 55 to 75 years, scheduled for surgical interventions of 1–4 h. Exclusion criteria were cardiac surgery and neurosurgery, patients who did not understand English, French or Dutch, and patients with visual/auditory impairments, chronic and acute infections, or inability to perform cognitive testing.

In practice, not excluded surgical types were categorized into general, genitourinary and orthopedic surgeries to individualize their possible influence on study endpoints. Overall, these inclusion/exclusion criteria were chosen to constitute a homogenous surgical patient population in which perioperative care could be standardized.

### Pre- and post-operative assessments

Prior to surgery, patients had a baseline Mini-Mental Status Examination (MMSE) assessment by a trained assessor. Relevant patient demographic information, including smoking and alcohol consumption, was collected. Data from the large version of the International Physical Activity Questionnaire (IPAQ) and the Geriatric Depression Scale (GDS) were also recorded for each patient. A peripheral blood sample was drawn to evaluate circulating IL-6 and HMGB1 levels (analyzed by ELISA) and used as inflammatory and DAMP markers. Six hours postoperatively, a peripheral blood sample was drawn again as well as 24 h postoperatively to re-evaluate inflammatory and DAMP markers. Patient MMSE was re-assessed 6 weeks and whenever possible 3 months after surgery by a trained assessor. The primary endpoint was defined as the change in MMSE score between baseline and 6 weeks post-surgery. The study particularly focused on the relationship between the 6-week change in MMSE and sedentary lifestyle as measured by the IPAQ recorded sitting time (h/day).

### Anesthesia management

Standardized anesthetic management included ECG, pulse oximetry, non-invasive blood pressure (every three minutes) and neuromuscular blockade monitoring (utilizing the train-of-four ratio). General anesthesia was induced with intravenous sufentanil (0.2 μg kg^− 1^), lidocaine (1–1.5 mg kg^− 1^) and propofol (2–3 mg kg^− 1^). Rocuronium (0.5–1 mg kg^− 1^) was administered to facilitate tracheal intubation. Additional 10–20 mg boluses of I.V. rocuronium were administered when necessary. Anesthesia was then maintained with 0.5–2.5% sevoflurane in an O_2_-air mixture, the latter being titrated to maintain oxygen saturation (SpO2) to a value of 96% or higher via pulse oximetry. Phenylephrine was used to maintain mean arterial blood pressure within 20% of the preoperative value. Acetaminophen (1000 mg) and diclofenac (1 mg kg^- 1^) were administered for analgesia in all patients. I.V. sugammadex (4 mg kg^− 1^) was administered to reverse neuromuscular blockade. After extubation, patients were placed in the post-anesthesia care unit, before returning to the ward.

### Statistical analysis

We hypothesize a relationship between sedentary lifestyle and PND in (pre-) elderly subjects undergoing surgery. A sample size calculation setting power at 80% and significance level at 5% showed that by enrolling at least 29 patients in the study, a correlation of 0.50 (25% of explained variance) could be evidenced between IPAQ sitting time and a drop in MMSE 6 weeks after surgery using a two-sided Student t test.

Results were summarized as mean and standard deviation (SD) for quantitative variables and as median and interquartile range (IQR) for skewed data. Frequency tables (number, percent) were used for categorical findings. Some variables were log-transformed (IL-6, HMGB1) or square root transformed (IPAQ items) to normalize their distribution and statistical analyses were done on the transformed data. The correlation coefficient was used to measure the association between two quantitative variables. Changes in MMSE scores between baseline and other time points (6w and 3 m) were assessed by the paired Student t test, and similarly for IL-6 and HMGB1 changes. To test the overall effect of baseline covariates on evolution of MMSE, IL-6 and HMGB1, data were also analyzed by linear mixed effect models. Time adjusted effects of covariates were then expressed as regression coefficients with standard error (SE); a positive (negative) coefficient would indicate an increasing (decreasing) impact of the covariate on the outcome. The statistical significance level was set at 5% (*p* < 0.05). Calculations and graphs were done with SAS (version 9.4) and R (version 3.6.1).

## Results

### Study conduct and patient baseline characteristics

Thirty-eight patients were included in the study. Their baseline characteristics are described in Table 1. In particular, the median IPAQ sitting time was 7 h/day (IQR: 6–9 h/day). Of the 38 study patients, 6 (15.8%) could not be evaluated after surgery.

**Table 1:** Baseline characteristics of study patients (*n* = 38)

Variable	Category	Mean ± SD Number (%)
Age (years)		64.8 ± 6.4
Sex	Female	16 (42.1)
	Male	22 (57.9)
BMI (kg/m^2^) (*n* = 37)		28.8 ± 5.7
Education level	< High school	15 (39.5)
	High school	17 (44.7)
	Undergraduate degree	4 (10.5)
	Graduate Degree	1 (2.6)
	Postgraduate degree	1 (2.6)
No. of languages known	1	20 (52.6)
	2	11 (28.9)
	3	6 (15.8)
	6	1 (2.6)
Working	No	30 (78.9)
	Yes	8 (21.1)
Marital status	Married	25 (65.8)
	Divorced	8 (21.1)
	Widow	5 (13.2)
Self-sufficient	No	3 (7.9)
	Yes	35 (92.1)
Living environment (No. of people)	1	12 (31.6)
	2	24 (63.2)
	3	1 (2.6)
	4	1 (2.6)
Number of alcoholic drinks/weeks	0	22 (57.9)
	1	6 (15.8)
	≥ 2	10 (26.3)
Smoking Status	Non-smoker	15 (39.5)
	Past smoker	14 (36.8)
	(Mean no. of years since quitting)	13.1 ± 12.2
	Current smoker	9 (23.7)
	(Mean no. of cigarettes/day)	12.2 ± 7.3
Surgery	General	12 (31.6)
	Genitourinary	15 (39.5)
	Orthopedic	11 (28.9)
No. of psychoactive drugs	0	32 (84.2)
	1	5 (13.2)
	2	1 (2.6)
Type of psychoactive drug (*n* = 6)	Benzodiazepines	5 (83.3)
	SSRI	1 (16.7)
Type 2 diabetes	No	28 (73.7)
	Yes	10 (26.3)
IPAQ work-related (h/week)		0.26 ± 0.92
IPAQ transport-related (h/day)		0.63 ± 0.76
IPAQ household-related (h/day)		0.42 ± 0.60
IPAQ leisure time-related (h/day)		0.19 ± 0.41
IPAQ sitting time (h/day)		7.3 ± 2.4
Energy (METs-min)		239 ± 242
GDS (0–30)		6.66 ± 4.43
MMSE (/30)		25.8 ± 4.19
IL-6 (pg/ml) (*n* = 31)		31.2 ± 33.9
HMGB1 (pg/ml) (*n* = 33)		38.9 ± 85.6

*BMI* Body mass index, *SSRI* Selective serotonin reuptake inhibitor, *MMSE* Mini-mental state examination, *IPAQ* International Physical Activity Questionnaire (long version), *IL* Interleukin, *HMGB1* High molecular group box protein 1, *METs* Metabolic Equivalents, *GDS* Geriatric depression scale

### MMSE and patient characteristics

As seen in Table 2, the mean MMSE score was 25.8 ± 4.2 at baseline and 23.6 ± 4.8 6 weeks after surgery. Based on the 32 patients who were seen at both visits, this corresponds to a significant decrease of 2.1 ± 3.1 points (*p* = 0.0006) or to an 8.2% drop from baseline. For the 19 patients whose MMSE was available 3 months after surgery, scores had gone up a little but tended to remain lower than baseline scores (*p* = 0.055). Regression analysis showed that the drop in MMSE score after 6 weeks (primary endpoint) was not related to daily sitting time (correlation coefficient 0.11, *p* = 0.55). Linear mixed model analysis on time-related MMSE data (Table 3) confirmed this finding (regression coefficient ± SE: − 1.75 ± 1.53, *p* = 0.26). It also evidenced that knowledge of several languages (*p* = 0.028), being self-sufficient (*p* = 0.0083) and good pre-operative MMSE score (*p* < 0.0001) were associated with overall greater postoperative MMSE scores. By contrast, high baseline GDS scores were indicative of lower MMSE scores (*p* = 0.0015). Of note, a positive tendency was found for education level (*p* = 0.069), active working status (*p* = 0.080), number of hours of work per week (*p* = 0.084) and number of hours of leisure time per day (*p* = 0.066); type 2 diabetes tended to act as worsening of MMSE scores (*p* = 0.068). All other covariates turned out to have no real effect on postoperative MMSE scores, in particular IL-6 and HMGB1 baseline levels or their respective increase up to 24 h post-operatively.

**Table 2:** Time-related evolution of MMSE in study patients

Variable	Time	Number. of patients	Mean ± SD	Median (IQR)
MMSE (0–30)	Baseline	38	25.8 ± 4.2	27 (23.0–30.0)
	6 weeks	32	23.6 ± 4.8	25 (20.0–27.5)
	3 months	19	24.5 ± 5.2	26 (21.0–29.0)
Drop MMSE	Baseline-6w	32	2.1 ± 3.1^(a)^	1.5 (0.5–4.0)
	Baseline-3 m	19	1.6 ± 3.4^(b)^	1.0 (0.0–3.0)

^(a)^ *p* = 0.0006 and ^(b)^*p* = 0.055 (both paired Student t-test)

*MMSE* Mini-mental state examination

**Table 3:** Effect of baseline parameters on the evolution of MMSE scores

Variable	Coefficient (SE)*	*P*-value
Age (years)	−0.071 (0.11)	0.53
Gender (Male vs. Female)	0.23 (1.41)	0.87
BMI (kg/m^2^)	−0.077 (0.13)	0.55
Education level^(a)^	1.36 (0.72)	0.069
No. of languages known	1.44 (0.63)	0.028
Working (Yes vs. No)	2.95 (1.64)	0.080
Marital status		
(Divorced vs Married)	−0.080 (1.75)	0.96
(Widow vs. Married)	0.65 (2.13)	0.76
Self-sufficient (Yes vs. No)	6.44 (2.31)	0.0083
Living situation (No. of people)	0.67 (1.10)	0.55
Number of alcoholic drinks/week^(b)^	0.48 (0.53)	0.37
Smoking Status		
(Current vs. Never)	−0.38 (1.52)	0.81
(Past vs. Never)	−3.50 (1.73)	0.51
Surgery		
(Orthopedic vs. General)	1.52 (1.76)	0.39
(Genitourinary vs. General)	−0.95 (1.64)	0.57
No. of psychoactive medications^(b)^	−0.91 (1.75)	0.61
Type 2 Diabetes (Yes vs. No)	−2.82 (1.50)	0.068
IPAQ work-related (h/week)^(b)^	2.49 (1.40)	0.084
IPAQ transport-related (h/day)^(b)^	0.78 (1.45)	0.59
IPAQ household-related (h/day)^(b)^	1.00 (1.39)	0.48
IPAQ leisure time-related (h/day)^(b)^	3.32 (1.75)	0.066
IPAQ sitting time (h/day)^(b)^	−1.75 (1.53)	0.26
Energy (METs-min)^(b)^	0.045 (0.083)	0.59
GDS	−0.47 (0.14)	0.0015
MMSE baseline	0.92 (0.067)	< 0.0001
IL-6 baseline (pg/ml)^(c)^	−0.31 (0.46)	0.50
IL-6 increase until 24 h (Yes vs. No)	1.47 (1.56)	0.35
HMGB1 baseline (pg/ml)^(c)^	0.13 (0.46)	0.77
HMGB1 increase until 24 h (Yes vs. No)	0.13 (1.76)	0.94

*covariate regression coefficients are adjusted for time; a positive (negative) coefficient is associated with an increasing (lowering) impact of parameter on MMSE scores over time

(a) treated as an ordinal variable

(b) square root transform applied to normalize the distribution

(c) log-transform applied to normalize the distribution

*BMI* Body mass index, *SE* Standard error, *MMSE* Mini-mental state examination, *IPAQ* International Physical Activity questionnaire (long version), *IL* Interleukin, *HMGB1* High molecular group box protein 1, *GDS* Geriatric depression score

### Inflammatory markers

**Fig. 1:**
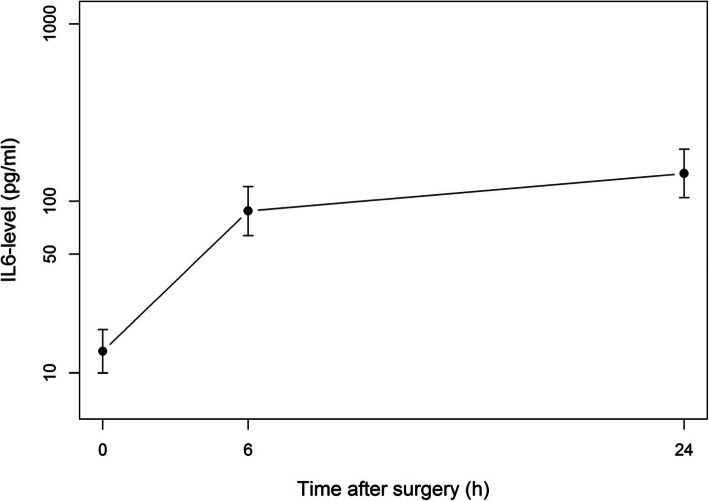
Evolution of IL-6 (pg/ml) levels after surgery

The distribution of IL-6 was highly skewed so data were log-transformed. The evolution of IL-6 is displayed graphically in Fig. 1. The median (IQR) IL-6 level rose from 23.5 (2.9–42) pg/ml at baseline to 138 (46.3–247) pg/ml 6 h after surgery (*p* < 0.0001). After 24 h, levels were still higher than those at baseline with a median level of 193 (86.8–528) pg/ml (*p* < 0.0001). Linear mixed model analysis applied to assess the effect of each covariate on IL-6 time-related levels (Table 4) showed that age (*p* = 0.0044) and baseline IL-6 value (*p* < 0.0001) impacted positively post-operative IL-6 levels. By contrast, the number of psychoactive drugs taken preoperatively (*p* = 0.041) and the number of hours of work per week (*p* = 0.024) were associated with lower IL-6 levels after surgery. No other covariate was found to be of interest.

**Table 4:** Effect of baseline parameters on the time evolution of IL-6 and HMGB1 levels

Variable	Coefficient (SE) on IL-6*	*P*-value	Coefficient (SE) on HMGB1*	*P*-value
Age (years)	0.13 (0.041)	0.0044	0.026 (0.033)	0.45
Gender (Male vs. Female)	0.75 (0.54)	0.18	0.30 (0.41)	0.47
BMI (kg/m^2^)	0.027 (0.049)	0.59	−0.060 (0.033)	0.079
Education level^(a)^	−0.27 (0.29)	0.35	−0.37 (0.21)	0.086
No. of languages known	−0.027 (0.25)	0.95	0.24 (0.19)	0.21
Working (Yes vs. No)	−0.79 (0.63)	0.23	0.15 (0.50)	0.77
Marital status				
(Divorced vs Married)	0.19 (0.63)	0.77	−0.25 (0.48)	0.60
(Widow vs. Married)	−0.85 (0.95)	0.38	−0.45 (0.64)	0.49
Self-sufficient (Yes vs. No)	−0.53 (1.10)	0.63	−0.22 (0.73)	0.77
Living situation (No. of people)	0.038 (0.42)	0.93	0.33 (0.31)	0.29
No. of alcoholic drinks/week^(b)^	−0.078 (0.22)	0.72	−0.024 (0.17)	0.89
Smoking Status				
(Current vs. Never)	−0.45 (0.63)	0.48	0.55 (0.45)	0.23
(Past vs. Never)	−0.73 (0.71)	0.31	0.79 (0.53)	0.15
Surgery				
(Orthopedic vs. General)	0.19 (0.67)	0.78	−0.39 (0.49)	0.44
(Genitourinary vs. General)	−0.80 (0.66)	0.23	−0.90 (0.48)	0.073
No. of psychoactive medications^(b)^	−1.25 (0.59)	0.041	−0.40 (0.47)	0.40
Type 2 Diabetes (Yes vs. No)	0.79 (0.64)	0.22	0.53 (0.45)	0.25
IPAQ work-related (h/week)^(b)^	−1.55 (0.65)	0.024	0.064 (0.55)	0.91
IPAQ transport-related (h/day)^(b)^	−0.36 (0.63)	0.57	−0.41 (0.44)	0.36
IPAQ household-related (h/day)^(b)^	−0.30 (0.53)	0.57	−0.65 (0.38)	0.092
IPAQ leisure time-related (h/day)^(b)^	−0.86 (0.66)	0.21	−0.093 (0.51)	0.86
IPAQ sitting time (h/day)^(b)^	0.058 (0.62)	0.93	−0.40 (0.43)	0.36
Energy (METs-min)^(b)^	−0.012 (0.032)	0.71	0.0030 (0.023)	0.90
GDS	0.039 (0.062)	0.54	−0.009 (0.045)	0.84
MMSE baseline	0.029 (0.074)	0.70	0.038 (0.051)	0.46
IL-6 baseline (pg/ml)^(c)^	0.86 (0.084)	< 0.0001	0.059 (0.14)	0.69
HMGB1 baseline (pg/ml)^(c)^	0.097 (0.18)	0.59	0.68 (0.065)	< 0.0001

*covariate regression coefficients are adjusted for time; a positive (negative) coefficient is associated with an increasing (lowering) impact of parameter on IL-6 or HMBD1 levels over time

(a) treated as an ordinal variable

(b) square root transform applied to normalize the distribution

(c) log-transform applied to normalize the distribution

*BMI* Body mass index, *SE* Standard error, *MMSE* Mini-mental state examination, *IPAQ* International Physical Activity questionnaire, *IL* Interleukin, *HMGB1* High molecular group box protein 1, *METs* Metabolic equivalents, *GDS* Geriatric depression scale

**Fig. 2:**
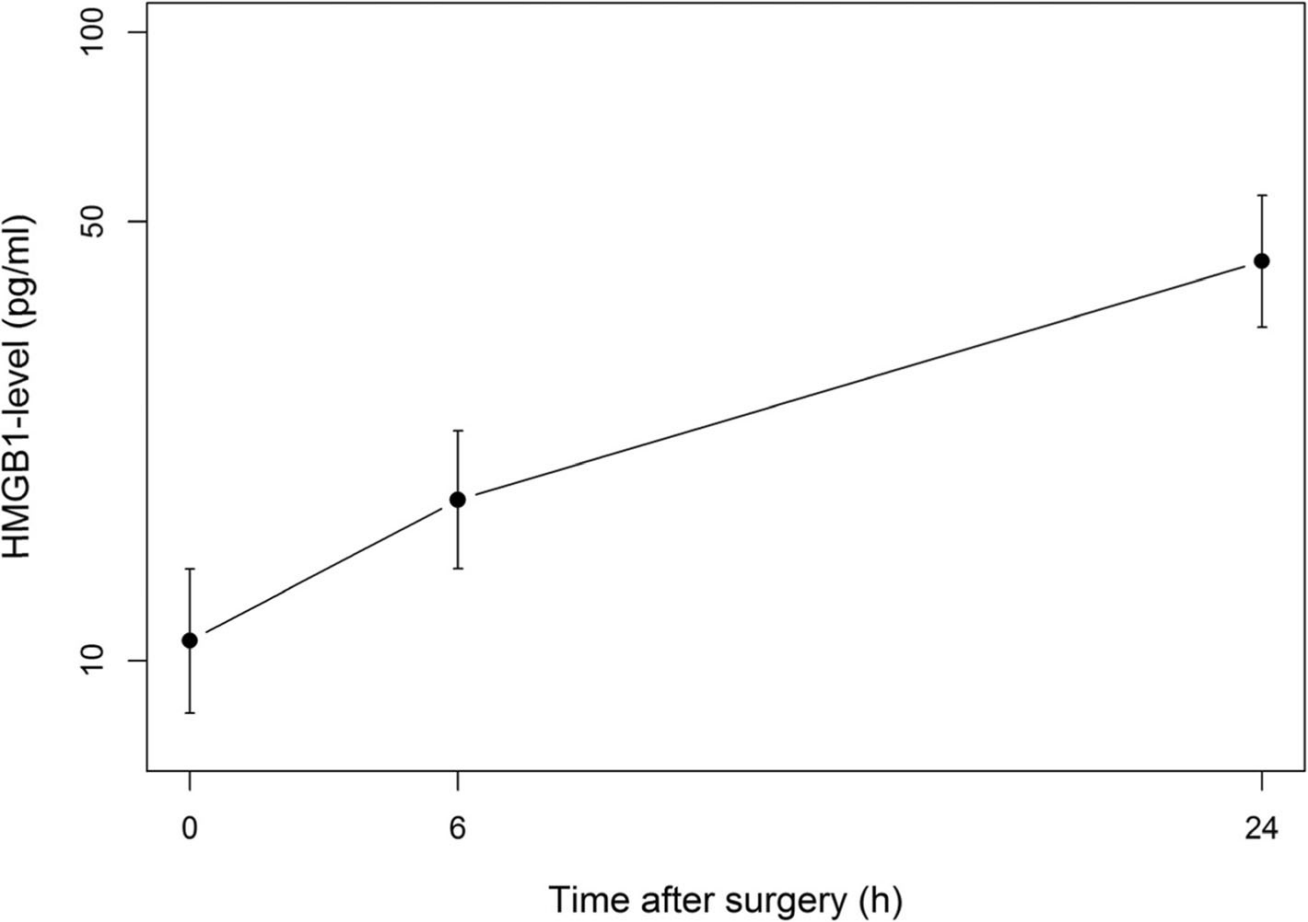
Evolution of HMGB1 (pg/ml) levels after surgery

HMGB1 levels were also log-transformed. Their evolution within 24 h after surgery is depicted in Fig. 2. The median (IQR) HMGB1 level increased from 8.53 (4.6–27.2) pg/ml at baseline to 19.9 (12.0–33.2) pg/ml 6 h after surgery (*p* = 0.0075). Until 24 h, HMGB1 levels continued to increase to reach a median level of 48.2 (24.4–75.6) pg/ml. When analyzing the relationship between each baseline covariate and post-operatives HMGB1 levels (Table 4), no association was found statistically significant, except for baseline HMGB1 levels (*p* < 0.0001), indicating that patients with higher level before surgery mostly kept high levels after surgery.

## Discussion

### Summary of findings

The present study indicates that at least 75% of non-cardiac surgical patients experienced a decrease in MMSE levels 6 weeks postoperatively with a highly significant mean drop of 2.1 ± 3.1 points (*p* = 0.0006). It also showed that a postoperative increase in IL-6 and HMGB1 levels was observed in all patients. Sedentary behavior expressed by the sitting time (h/day) is neither a risk factor for PND nor for postoperative peripheral inflammation and DAMP. In contrast to environmental factors, constitutive factors influenced MMSE scores and hence PND. Thus, patients who were self-sufficient and scored lower on the GDS had higher MMSE scores. Similarly, patients speaking multiple languages had better MMSE scores. Postoperative rise in IL-6 was influenced by age, number of psychoactive drugs taken by the patient and type of surgery. IL-6 levels were lower in patients with higher work-related IPAQ scores. Trauma-induced HMGB1 was not influenced by demographic or environmental characteristics.

### Modifiable risk factors

Lifestyle behavior has been advanced as a modifiable risk factor in the prevention of PND development [15]. Regarding baseline covariates, sedentary behavior is neither a risk factor for PND nor is it associated with an increase in peripheral inflammation in the elderly surgical patient. This finding contrasts with an earlier preclinical study which demonstrated that postoperative cognitive decline was higher in low capacity runner rats; preoperative exercise reversed the vulnerability for cognitive decline [12]. Similarly, a clinical study by Hudetz et al. demonstrated that patients with metabolic syndrome experienced reductions in tests of verbal memory and executive function and overall cognitive performance after surgery [17]. While sedentary behavior contributes to metabolic syndrome, the patients in our study did not necessarily suffer from this syndrome. This could perhaps explain the different results observed between this study and previous clinical and preclinical studies [12, 16–18].

### Constitutional risk factors: Inflammaging

Aging is associated with immune dysregulation, of which the most evident characteristics are higher circulating levels of pro-inflammatory cytokines. Inflammaging is thought to contribute to many of the diseases of the elderly, such as infections, autoimmune disorders, and chronic inflammatory diseases [21, 22]. A study by Cohen et al. showed the correlation between serum IL-6 levels and age [23]. Similarly, in this study, postoperative IL-6 increase was also influenced by age. Age (> 60 years old) has been suggested to be a risk factor for the development of PND [5, 17]; several studies confirm this association [24, 25].

### Surgery-associated HMGB1 release

Surgery is associated with an increase in HMGB1, a well-known DAMP, in preclinical and clinical studies [26–28]. Similarly, in our study, HMGB1 levels increased postoperatively, regardless of age. Age-related inflammation, measured by baseline IL-6, did not correlate with these HMGB1 levels (*p* = 0.69). Preclinical studies have shown that disabling HMGB1 leads to lowering systemic and hippocampal inflammatory responses to surgery and prevents the development of PND [7, 29]. This study demonstrates that while IL-6 levels were influenced by environmental and constitutive factors, this was not the case for the trauma-induced release of HMGB1.

### Limitations

This study both has and reveals some limitations. Firstly, at present, a consensus for neuropsychological testing tools to diagnose PND does not exist. MMSE, the cognitive testing tool used in this study, is widely accepted and used in clinical studies examining the incidence of PND because of its familiarity and ease of administration. However, it may be criticized as a cognitive diagnostic tool as it lacks the sensitivity and specificity to detect subtle cognitive impairment and it is limited by both floor and ceiling effects [30, 31]. Ideally, until a consensus is reached regarding the exact testing methods, a battery of cognitive tests should be used to diagnose PND. Nonetheless, in the present study, MMSE variations under constitutive and environmental factors have provided emerging clues for future studies.

Secondly, though exercise and sedentary behaviour are opposite sides of the same coin (degree of motor activity), they should be considered as two distinct entities. Previous work showed that, in a preclinical setting, the active introduction of exercise was associated with less post-operative cognitive dysfunction [32]. Our present study evaluates the impact of pre-existing sedentary behavior on perioperative cognitive dysfunction; however, as the effect of exercise on outcome was not measured we cannot assume that its effects will be the polar opposite of sedentary behavior.

Thirdly, in this study, peripheral cytokines were analyzed, examining only one part of the inflammatory cascade leading to PND. Large randomized controlled trials with peripheral serum and cerebrospinal fluid (CSF) samples are needed to further examine this inflammatory cascade. Lastly, the results of this study are based on the analysis of a limited sample of patients.

## Conclusion

Surgery is associated with an increase in peripheral IL-6 and HMGB1 and with cognitive impairment 6 weeks postoperatively. Preoperative sedentary behavior is `neither a risk factor for PND nor is it associated with an increase in peripheral inflammation, findings that correspond with pre-clinical data.

## Data Availability

The datasets used and/or analyzed during the current study are available from the corresponding author on reasonable request.

## Abbreviations

BM-DM: Bone marrow-derived monocytes
DAMP: Damage-associated molecular pattern
ELISA: Enzyme-linked immunosorbent assay
GDS: Geriatric Depression Scale
HMGB1: High molecular group box protein
IL-1 beta: Interleukin 1 beta
IL-6: Interleukin 6
IPAQ: International Physical Activity Questionnaire
IQR: Interquartile range
MMSE: Mini-mental state examination
PND: Perioperative neurocognitive disorders
SD: Standard deviation
SE: Standard error
